# Dietary phenolics and exercise complementation to delay aging at its source: a comprehensive review highlighting mitochondrial function

**DOI:** 10.3389/fragi.2025.1693043

**Published:** 2025-12-19

**Authors:** Jiong Zhang, Wen-Wen Zhu, Yong-Yao Huang, Chuan-He Tang

**Affiliations:** The Research Group of Food Colloid and Nanotechnology, School of Food Science and Engineering, South China University of Technology, Guangzhou, China

**Keywords:** aging, mitochondria, dietary phenolics, exercise, combined anti-aging

## Abstract

**Background:**

Currently, aging issues are becoming more prominent, and the aging population is expanding. The reliance on medical or pharmaceutical means of combating aging and disease raises concerns about the long-term safety and economic impact. Therefore, sustainable and friendly strategies need to be explored urgently. Phenolic-rich antioxidant dietary regimens and exercise integrated into daily habits contain great anti-aging potential. Research on natural laws for anti-aging based on phenolics and exercise is in full swing.

**Scope and approach:**

The review first outlines the current status of aging and elucidates the root causes of aging. Second, the anti-aging mechanisms at the source through daily behaviors such as phenolic diets and exercise are introduced. Then, the combined anti-aging strategy of dietary phenolic supplements and exercise is proposed, providing a feasible basis for resource synergy between the two. Finally, constructive comments are made to guide practical implementation and future development.

**Key findings and conclusions:**

Mitochondrial dysfunction and its ROS accumulation are the essence of aging pathogenicity, and its causes include lifestyle habits, age, and genes. The precise action on mitochondria through phenolics and exercise to ameliorate oxidative stress and maintain anti-aging function is in line with contemporary concepts of anti-aging. Although research on the combined effects of phenolics and exercise for anti-aging is scarce and faces multiple challenges, this new strategy is likely to be adopted as these issues are gradually resolved.

## 1 Introduction

**FIGURE 1:**
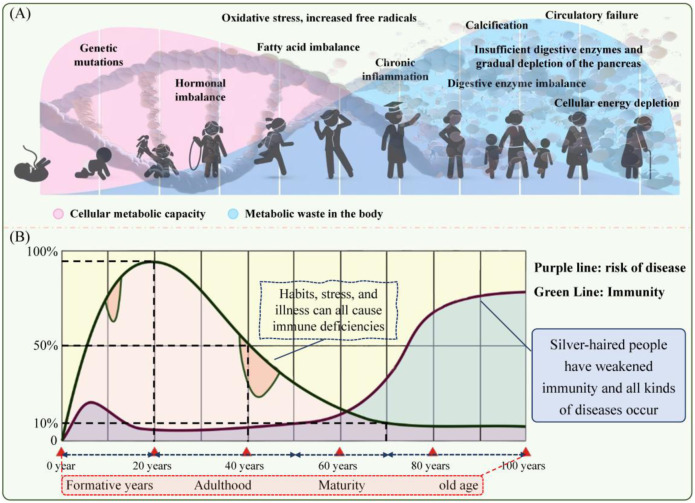
Natural and complex aging process **(A)** and the aging–immunity–disease risk relationship **(B)**.

Against the macro backdrop of diverse coexistence and rapid development, the onset of the “silver wave” is a distinctive global trend of our time. According to the World Health Organization statistics, by 2050, the proportion of the world population over 60 is expected to increase from 12% to 22%, reaching 2.1 billion (WHO INT, 2025). In addition, the current state of aging will continue to intensify. Aging is a slow and gradual process that begins in the cell and is influenced by genetics and the environment (Figure 1). In biological terms, aging is the result of an ongoing accumulation of molecular and cellular stochastic damage at every level over time, manifesting in the decay of structure, function, adaptability, and resistance; increased probability of disease; and aggravated risk of death (Cai et al., 2022; Sen et al., 2016). Improvements in overall healthcare have led to longer life expectancy than in the past; however, healthy life years have remained largely unchanged, meaning that the extra years are accompanied by poor conditions (Behr et al., 2023). More than 75% of people over 60 years suffer from one or more chronic conditions because of aging (Beard et al., 2016; Zhang et al., 2020). Aging-related issues affect almost all areas of society, such as politics, the economy, livelihoods, and family life. It is not only the elderly who are affected—many individuals aged 20–40 years have a biological age that is 10–15 years higher than their chronological age, illustrating the phenomenon of “premature aging” (Felix et al., 2024).

With regard to aging, the most famous hypothesis that is supported by a large body of modern scientific evidence to explain it is the free radical theory proposed by Harman (1956). The essence is the oxygen radical theory of aging, which postulates that the assault on the body by the imbalanced production and neutralization of ROS triggers oxidative stress, which disrupts the inherent order and causes aging. ROS are respiratory by-products of all known aerobic organisms, and mitochondria are the first production and key scavenging sites for ROS (Boengler et al., 2017). Therefore, mitochondria are rightfully thought to be closely related to aging. As observed by Boengler et al. (2017), aged heart, skeletal muscle, and adipose tissue exhibited mitochondrial dysfunction characterized by increased ROS formation. This is why mitochondrial dysfunction has long been listed as one of the hallmarks to indicate aging (Kroemer et al., 2025; Lopez-Otin et al., 2013). In the ongoing exploration of the mechanisms of aging, scholars have been surprised to discover that mitochondria also play a central role, thereby advancing the theory further.

Taking sleep as an example, Vaccaro et al. (2020) observed high levels of ROS in flies and mice subjected to sleep deprivation. They noted that if ROS could be effectively removed, even animals kept awake for extended periods had lifespans comparable to those maintaining a normal sleep routine. In summary, excess ROS in mitochondria is considered a pathobiochemical mechanism that progresses from cellular aging to disease onset. Therefore, correcting mitochondrial dysfunction through antioxidants, waste removal, revitalization, or scavenging cells as pathways is both consistent with the updated definition of disease by the World Health Organization and the essence of health innovation. Various interventions have been interpreted and used to balance mitochondria, such as epigenetic modulation (Wu et al., 2024), organ transplantation (Nakai et al., 2024), infusion of stem-cell populations (Iorio et al., 2024; Zhang and Miao, 2023), enzyme replacement (Rubilar et al., 2024), physical activity, caloric restriction, dietary modifications including increased intake of antioxidant foods and supplementation with specific nutrients (Sharma and Mehdi, 2023), and the use of drugs targeting conservative longevity pathways, such as metformin and rapamycin (Amorim et al., 2022). In line with the global priority of anti-aging and the initiatives of the Decade of Action for Healthy Aging, and given the high cost, uncertainty, and possible side effects of medicinal treatments, proactive antioxidant nutritional changes and exercise are the best option to build health from endogeny.

After analyzing the association between long-term adherence to healthy dietary patterns and aging in two large prospective cohorts in the United States over more than 30 years, Tessier et al. (2025) reported that higher intake of fruits, vegetables, whole grains, nuts, legumes, and unsaturated fats were associated with healthy aging (defined as reaching 70 years without 11 major chronic diseases and with preserved cognitive, physical, and mental functioning). The high percentage of bioactive constituents is a salient feature of the listed ingredient profile in the form of unsaturated fatty acids, phenolics, fibers, vitamins, and minerals, with phenolics demonstrated to be the dominant agonist. Phenolics, which are synonymous with antioxidants, are widely found in numerous daily food resources such as fruits, vegetables, tea, wine, coffee, whole grains, legumes, nuts, and spices (Avila-Roman et al., 2021; Lin et al., 2024). Earlier, different *in vitro*, *in vivo*, and clinical trials revealed that phenolics/phenol-rich diets have the full spectrum of activities of antioxidant, anti-inflammatory, anti-“three highs,” cardiovascular protection, and intestinal flora regulation, which can affect functions ranging from minor illnesses to cancer (Gai et al., 2023; Wu et al., 2021). This is the health code for people in the “blue zone,” in contrast to inadequate intake of fruits, vegetables, and whole grains, along with diets high in fats and oils (Fang et al., 2023). The advantages of low cost and availability of phenolics have advanced their kernel theory research. Pieces of evidence continue to confirm that phenolics rescue mitochondrial function through multiple abilities, such as scavenging free radicals, rectifying the enzyme system, stimulating the endogenous production of essential substances, regulating quality control processes, and interacting with the gut flora, which is exciting and lays the groundwork for their use in anti-aging processes.

On the other hand, exercise has long been recognized for inducing humoral factors such as peptides, metabolites, DNA, and RNA to modulate the bodily inflammatory state for youthfulness (Safdar et al., 2016). The life clocks, such as the plasma proteomics, the Horvath, the Hannum, the PhenoAge, and the GrimAge, all reflect the correlation between physical activity and age (Felix et al., 2024). The biological age of those who exercised consistently is 5.8 years younger than that on their ID cards (Argentieri et al., 2025). In recent years, physical activity has been comprehensively reported to enhance mitochondrial homeostasis through pleiotropic mechanisms that prevent aging and disease episodes. Unfortunately, current studies on phenolics and exercise for anti-aging have been conducted in isolation. Therefore, this review first deconstructs the nature of human aging and its intrinsic connection with mitochondria. Subsequently, the anti-aging mechanisms of phenolic diets and active exercise are elucidated from the long-term safety and sustainability perspective. Based on the high activity and low bioavailability of phenolics, adherence to the implementation of the “phenolic supplementation plus exercise” paradigm is advocated for overcoming aging. Finally, the application of both combination therapies is critically revisited, and constructive suggestions are made to address the existing issues and challenges of pharmacokinetic optimization, universality, and personalized protocol design.

## 2 Literature retrieval

**FIGURE 2:**
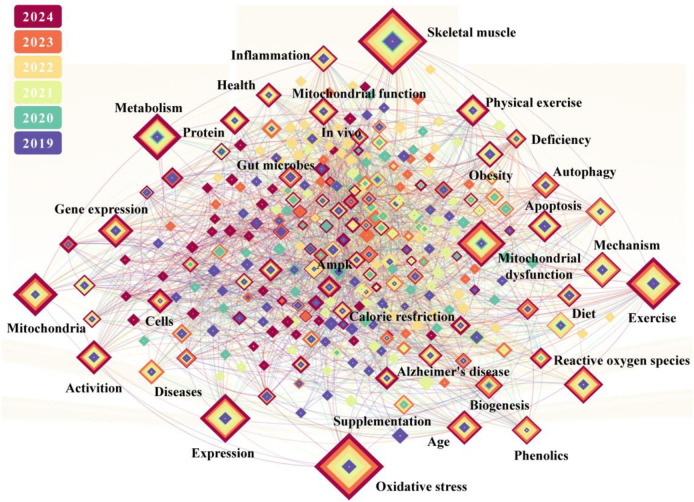
Correlation diagram of keywords for papers (retrieved from www.webofscience.com).

Figure 2 shows the articles published in recent years using the Web of Science Core Collection as the search database; the Science Citation Index Expanded as the edition; and aging, mitochondria, diet, exercise, and phenolic or polyphenol as the keywords. This retrieval adheres to the Preferred Reporting Items for Systematic Reviews and Meta-Analyses guidelines, and the 748 results are visualized using CiteSpace. It is clear that research on mitochondria has focused on exploring the important functions (including autophagy and apoptosis), identifying triggers of dysfunction (e.g., oxidative stress, inflammation, and gene expression), and adverse consequences (particularly the elevated susceptibility to neurodegenerative disorders exemplified by Alzheimer’s disease and metabolic syndromes typified by obesity). The effects and mechanisms by which dietary or physical activity-based strategies improve mitochondrial dysfunction are also delineated across three key domains: skeletal muscle plasticity, caloric restriction-related pathways, and cellular metabolism. However, studies on phenolics and exercise remain relatively independent. Their synergy is not reflected in the major keyword clusters related to aging. In the Web of Science database, searches using the keywords “phenolics” and “exercise” indicate that their intersection is limited to studies examining the impact of phenolics on exercise performance. This requires incorporating and integrating phenolics and exercise with biology to empower the great health industry based on the multi-omics-driven discovery, provide comprehensive health solutions for humans, and promote transformative innovation in global health.

## 3 Mitochondria and health: the truth behind aging

**FIGURE 3:**
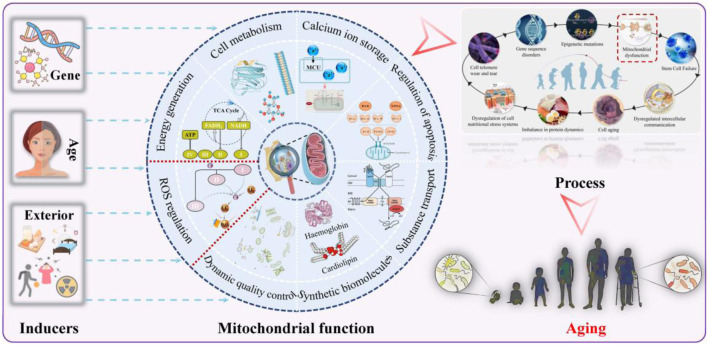
Mitochondrial function, causes of their impairment, and implications for aging.

As shown in Figure 3, mitochondria are important membrane structures in eukaryotic cells, primarily responsible for energy production and ROS regulation, and are also involved in metabolic processes, calcium ion storage, and apoptosis control (He et al., 2025). ROS in mitochondria usually originate from (1) “electron leakage” during electron transport through respiratory chain complexes Ⅰ and Ⅲ; (2) the proton gradient surge across the mitochondrial membrane; (3) lipid peroxidation of polyunsaturated membranes in macrophages and mitochondria dominated by enzymatic systems including NADPH oxidases and peroxidases; and (4) external stresses (Hong et al., 2024; Tang et al., 2017). Appropriate ROS can orchestrate a sophisticated division of labor across energy production, cellular remodeling, signal transduction, and immune defense (Qin et al., 2025; Ryu et al., 2024). Under normal conditions, mitochondria can autonomously respond to sudden ROS variations through efficient repair and quality-control mechanisms involving biogenesis, fusion–fission dynamics, autophagy and antioxidant defenses.

### 3.1 Antioxidant system

First, the body has evolutionarily developed a comprehensive antioxidant defense system accordingly, consisting of endogenous antioxidants such as SOD, CAT, and GPX and exogenous antioxidants mainly derived from dietary sources such as phenolics and vitamin C. Mitochondrial ROS stabilize the Nrf2 signaling axis to upregulate the transcription of endogenous antioxidant enzymes that initiate strong antioxidant activities and execute ROS scavenging (Manford et al., 2020). Second, exogenous antioxidants use a sacrificial mechanism by pre-emptively neutralizing free radicals to protect cells and the organism.

### 3.2 Quality-control network

Specifically, mitochondrial biogenesis is master-regulated by PGC-1α, which enhances TFAM expression through coordinated AMPK and SIRT1 signaling to integrate the cellular energy status (Abu Shelbayeh et al., 2023). Fusion and fission are controlled by the dynamic equilibrium of MFN1/2, OPA1, and DRP1, respectively, which coordinate energy fluctuations and stress signals to maintain functional integrity and eliminate damaged parts (Song, Ghochani et al., 2009). Autophagy can eliminate damaged mitochondria through both ubiquitin-dependent (acute: PINK1/Parkin pathway) and ubiquitin-independent mechanisms (chronic or sub-lethal stress: autophagy receptors such as BNIP3 and NIX directly interacting with LC3 *via* LIR motifs), thereby ensuring mitochondrial quality control and renewal. Severe ROS can be handled by nonselective autophagy, and mild ROS triggers selective autophagy dependent on mitochondrial fission (Redza-Dutordoir and Averill-Bates, 2021). These processes work in conjunction with each other to maintain or re-establish mitochondrial redox stability.

The outcome markedly diverges when mitochondrial function is abnormal (as manifested by reduced ATP, increased ROS, reduced NAD^+^ availability, diminished mitochondrial dynamics, impaired autophagy, and DNA mutations) due to progressive failure. They lose the ability to manage ROS, and ROS production (oxidation) and scavenging (anti-oxidation) are biased toward pro-oxidation, which is collectively defined as mitochondrial oxidative stress. “Mitochondrial dysfunction–oxidative stress” is a complex process. On one hand, ROS that cannot be scavenged in time can oxidize various macromolecules such as proteins, lipids, and DNA; depolarize the mitochondrial membrane potential, enabling more high-energy electrons to bind to oxygen; accelerate telomere shortening; and propagate senescence to surrounding healthy cells. On the other hand, the above results lead to neutrophil inflammatory infiltration, weakened stem cell regeneration, and pathological protease hypersecretion, further attacking mitochondria and other organelles and aggravating oxidative damage. The long-term exposure and accumulation of excessive ROS and the vicious cycle culminate in tissue aging and multi-system collapse, which, in turn, evolve into a series of complications such as diabetes, muscle disorder, osteoporosis, cardiovascular diseases, neurodegenerative diseases, and rare genetic diseases (Chen et al., 2023; Konig and McBride, 2024; Marchi et al., 2023). It follows that mitochondria serve as hubs for information perception, integration, and communication, with their dysfunction linking multiple hallmarks of aging through redox-sensitive signaling cascades. After examining mitochondrial changes within cells and their effects, it is concluded that signs of cellular senescence all stem from mitochondrial aging and intrinsic ROS production, consistent with the perspectives of Son and Lee (2021) and Martini and Passos (2023).

Examining the causes, the common narrative tends to blame aging and genetics for declines in health; although they are certainly an aspect of increased risk, they are one-sided. Argentieri et al. (2025) tracked 500,000 people over 5 years and subversively reported that life circumstances shape 77% of life differences, rather than the innate DNA code. In other words, bad life environments (pollution and radiation), styles (sedentary behavior, prolonged lying down, improper diet, and poor sleep), and social pressure will stimulate ROS generation that reduces the mitochondrial number, size, and function to varying degrees and increases the susceptibility of mitochondria to aging and fragmentation over time, which is the nature of contemporary aging that is more dangerous than age and genes (Kopp, 2024). Stamatakis et al. (2025) quantified the results of nearly 60,000 middle-aged and older adults, indicating that small changes in diet and exercise can significantly increase healthy life expectancy. A Chinese cohort study unexpectedly found that adherence to these healthy lifestyles can offset genetic risk, whatever the aging genotype (Ren et al., 2025). What do the repeated key messages teach us? In the absence of proper therapies for mitochondrial dysfunction, nutrition and exercise are not merely supportive or adjunctive approaches but rather the current first-line therapies, reinforcing the recommendation to use extrinsic “natural laws” to optimize mitochondria, enhance cellular fuction, and prevent intrinsic aging.

## 4 Mitochondrial aging intervention based on phenolics

**FIGURE 4:**
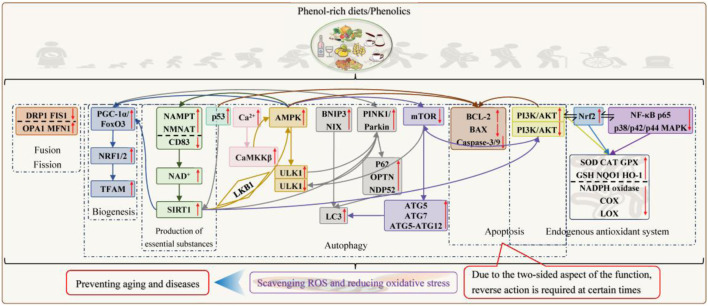
*In vivo* pathways for the elimination of oxidative stress by phenolics.

With improvements in food quality and nutrition, phenolics have begun to be emphasized and studied scientifically. The information on the classes, sources, structural characteristics, physicochemical properties, extraction methods, and biological activities of phenolics is presented in Table 1. The modulation of aging, lifespan, and metabolic burden (Calubag et al., 2024; Li et al., 2024a) and the epistatic direct effects on aging-related disease prevention and treatment with phenolics/phenolic diets (Arruda et al., 2020) have been validated based on animal models and clinical trials. A wide range of phenolics is mentioned, including but not limited to resveratrol, catechins, quercetin, anthocyanins, proanthocyanidins, curcumin, caffeic acid, chlorogenic acid, and lesser-known phenolics. With the growing understanding of the effects of mitochondria and phenolics on aging, the deeper underlying mechanisms and scientific principles are gradually being uncovered (Figure 4).

**TABLE 1:** Basic introduction to common phenolics.

Classification	Representative substance	Dietary source	Structural characteristic	Property	Extraction method	Biological activity	References
Phenolic acid	Gallic acid	Tea and pomegranate	Benzoic acid derivatives	Relatively stable; soluble in hot water and ethanol	Hot water extraction; alkaline solution extraction; microwave-assisted extraction	Antioxidant Antibacterial Anti-inflammatory Antiviral Anticancer Obesity control Heart protection	Jakobek and Blesso (2024), Favari, et al. (2024), Matsumura, Kitabatake, Kayano, and Ito (2023), and Rocchetti, et al. (2022)
	Caffeic acid	Coffee, fruits, and vegetables	Cinnamic acid derivatives	Relatively stable; soluble in hot water and ethanol	Enzyme-assisted extraction		
	Ferulic acid	Whole grains and coffee	Cinnamic acid derivatives	Relatively stable; soluble in hot water and ethanol	Enzyme-assisted extraction		
	Chlorogenic acid	Fruit and coffee	Caffeic acid derivatives	Relatively stable; soluble in hot water and ethanol	Water extraction; ultrasound-assisted extraction; supercritical fluid extraction		
	Coumaric acid	Soybeans and peanuts	Cinnamic acid derivatives	Relatively stable; soluble in hot water and ethanol	Enzyme-assisted extraction		
Flavonoid	Catechin	Green tea and cocoa	π–π conjugation	Unstable; soluble in hot water and ethanol	Hot water/ethanol extraction		
	Quercetin	Onions, apples, and berries	π–π conjugation	Unstable; insoluble in water	Organic solvent extraction; ultrasonic-assisted extraction		
	Anthocyanin	Berries	π–π conjugation	Unstable; easily soluble in water	Solvent extraction; ultrasonic extraction; microwave-assisted extraction		
	Kaempferol	Grapes and broccoli	π–π conjugation	Unstable; slightly soluble in water	Water or aqueous ethanol extraction		
	Daidzein	Soybeans and soy products	π–π conjugation	Relatively stable; soluble in water	Solvent extraction; fermentation-based release		
Diphenylethylene	Resveratrol	Grapes, red wine, and peanuts	π–π conjugation	Unstable; slightly soluble in water	Organic solvent extraction; solid-phase extraction		
Lignans	Lignans	Flaxseeds and grains	π–π conjugation	Unstable; insoluble in water	Solvent extraction		

### 4.1 Underlying logic of phenolics in managing mitochondrial aging

#### 4.1.1 Reduction of free radical production in mitochondria

Initially, fundamental investigations were conducted on phenolics, mitochondrial oxidation, and their oxidative stress markers. The results showed that quercetin, caffeic acid, curcumin, and resveratrol inhibited or quenched free radicals formed in mitochondrial respiratory chain complex Ⅲ in isolated rat hearts to different extents (Dudylina et al., 2019); foods and supplements enriched with resveratrol, chlorogenic acid, epicatechin, rutin, and ferulic acid have superoxide anion removing and chelating Fe^2+^ activity, which helped prevent liver mitochondria oxidative toxicity (Castro et al., 2023); rutin exhibited potent antioxidant activity against brain cell mitochondria in rats with aluminum-induced neurotoxicity, making it a promising candidate for neuroprotective agents (Kessas et al., 2024); the combination of chlorogenic acid and cinnamaldehyde oxidatively assassinated breast cancer cells by targeting mitochondria (Schuster et al., 2022). They neutralize mitochondrial free radicals through multifaceted mechanisms, including blocking chain reactions/propagation by electron or hydrogen atom supply, chelating metal ions that disrupt electron transport chain function, undergoing Fenton reactions, suppressing antioxidant defense systems, and inactivating free radical precursors. According to relevant research findings, 1 mg of tea polyphenols has a free radical scavenging capacity equivalent to 9 µg of SOD, and it is additionally 18 times more effective than vitamin E (Singh et al., 2010; Tang et al., 2019).

#### 4.1.2 Promotion of antioxidant enzyme production and inhibition of oxidase assembly

In addition to direct removal, phenolics can modify redox-sensitive thiol groups on Keap1 to dissociate the Nrf2–Keap1 complex. The liberated Nrf2 translocates to the nucleus, where it binds to antioxidant response elements in promoter regions. This action initiates gene transcription to upregulate the expression of downstream enzymes such as SOD, CAT, GPX, NQO1, or HO-1 to strengthen the construction of mitochondrial antioxidant capacity, as demonstrated by phenolics such as ferulic acid (Wang et al., 2021), quercetin (Liu Y. et al., 2024), theaflavin (Li et al., 2021), curcumin, allicin (Han et al., 2025), anthocyanins, resveratrol, and phenolic acids (Hussain et al., 2016), while they inhibit NADPH oxidase, NADH ubiquinone oxidoreductase, COX, and LOX assembly, which benefited rat hepatocyte mitochondria damaged by aflatoxin B1, brain cell mitochondria suffering from Alzheimer’s disease, kidney cell mitochondria with ischemia/reperfusion, and normal cell mitochondria. New evidence from studies on chlorogenic acid (Chen J.et al., 2024), curcumin (Sharma et al., 2022), piperine (Guo et al., 2025), and hydroxytyrosol (Zhang et al., 2021) suggests that phenolics can modulated the above-mentioned enzymes by inhibiting inflammatory pathways such as NF-κB p65 and p38/p42/p44 MAPK to reduce pro-inflammatory cytokine expression as well as up-regulating key signals such as PI3K/AKT either alone or in the synergistic crossover with the Nrf2 pathway. Moreover, the phenolic hydroxyl group of phenolics can provide electrons to stabilize metal ions (e.g., Cu/Zn) in the active center of SOD and enhance its catalytic efficiency.

#### 4.1.3 Stimulation of endogenous production of essential substances

It is well-known that biomolecules such as NAD^+^, coenzyme Q10, α-lipoic acid, and L-carnitine are essential endogenous agents for controlling ROS and sustaining mitochondrial activity, and they also have the potential to improve stem cell functionality (Chini et al., 2024; Mollazadeh et al., 2021). As a result, exogenous supplementation with these compounds and their precursors has been identified as a leading-edge strategy to reshape mitochondrial rejuvenation, but it faces the challenges such as inefficacy and antigenic problems with long-term use. In contrast, exogenous supplementation of phenolics can enhance intracellular levels of these substances and stimulate their activity. Phenolics having the driving force to enhance NAD^+^ conversion were first elucidated as early as 15 years ago through the resveratrol trial (Nature, 2010). In recent years, studies on tea polyphenols (Tribble et al., 2024), proanthocyanidins (Wan et al., 2021), and apigenin (Kramer and Johnson, 2024) have demonstrated the generalizability of the pioneering finding and established mechanistic links to sleep and retinal pathology models. The principle is that phenolics activate NAMPT and NMNAT to promote NAM recirculation and inhibit CD83 to reduce NAD^+^ consumption. The pro-generative effects of phenolics on coenzyme Q10, α-lipoic acid, and L-carnitine have not been examined, but they can indirectly modulate the levels of all three by scavenging free radicals and stabilizing the mitochondrial membrane to reduce oxidative depletion of coenzyme Q10, accumulation of oxidized α-lipoic acid, and exocytosis of L-carnitine. Starting with precursors and production enzymes will be a further direction to explore whether phenolics stimulate their direct production intracellularly.

#### 4.1.4 Improvement of the mitochondrial quality-control system

When discussing mitochondrial quality control, caloric restriction is an essential keyword, and the origins of the two begin in budding yeast, elegans, and mammals (Amorim et al., 2022). With subsequent research elucidating its mechanisms, the scientific community has established this strict life-long regimen as the most effective mitochondrial dietary intervention, providing dual benefits in managing metabolic disorders and aging (Russo et al., 2025). Davinelli et al. (2020) introduced phenolics to determine the relationship between caloric restriction and mitochondrial turnover equilibrium and suggested that phenolics may represent an interesting source of caloric restriction mimics. It is also known that phenolics such as tea polyphenols, proanthocyanidins, soy isoflavones, resveratrol (Chen S. et al., 2024), curcumin (Du et al., 2022), lithocholic acid (Qu et al., 2024), and baicalin (Wang T. et al., 2025) can participate in mitochondrial biosynthesis and energy metabolism transcription by activating PGC-1α/FoxO3 and downstream genes NRF1/2 and TFAM to improve insulin resistance, and aging-related phenotypes remodel the skeletal muscle fibers and extend the lifespan. The signaling pathway is divided into three parts: 1) direct activation of PGC-1α; 2) activation of SIRT1 for deacetylation of PGC1-α and FoxO3 by increasing intracellular NAD^+^ levels; and 3) activation of AMPK by depletion of ATP or direct phosphorylation of AMPK to increase NAD^+^ levels or phosphorylation of transcription factors such as FoxO3. According to Chen et al. (2018), EGCG ameliorated mitochondrial kinetic damage after subarachnoid hemorrhage by regulating DRP1, FIS1, OPA1, and MFN1 expression to control fission and fusion. Quercetin also protected alcoholic hepatocytes by reducing mitochondrial ROS excess and maintaining homeostasis through similar processes (Zhao et al., 2022). Gherardi et al. (2025), Gu and Han (2023), and Liu H. et al. (2025) elucidated the molecular mechanism by which phenolics regulate mitochondrial autophagy using resveratrol, curcumin, quercetin, EGCG, and oleuropein as examples. In short, phenolics facilitate the opening of two classical autophagy pathways by upregulating the expression of PINK1 and Parkin to elevate the levels of downstream autophagy markers (such as p62, OPTN, and NDP52) and bind to LC3 to enhance the degradation of ubiquitylated substrates (such as VDAC1 and MFN1/2) and by relying on the direct interaction of BNIP3 or NIX with LC3. Unexpectedly, phenolics also activate SIRT1 and SIRT3 (only present at low concentrations of 1 μM–5 μM) to potentiate the expression of PINK1/Parkin or PGC-1α or the deacetylation of FoxO1 and FoxO3 to ultimately enhance mitochondrial autophagy. Phenolics also activate AMPK by increasing intracellular Ca^2+^ levels to activate CaMKKβ, inhibiting mitochondrial ATP synthase, and deacetylating the kinase LKB1 by SIRT1 (low doses are dependent on SIRT1, while high doses are independent, but AMPK itself can enhance SIRT1 activity by increasing fatty acid oxidation and NAMPT expression to promote NAD synthesis), which can phosphorylate ULK1, initiate the formation of autophagosomes, activate Parkin, and regulate the mitochondrial autophagy process. In the context of mitochondrial biogenesis, phenolics promote the interaction between AMPK and PGC-1α, facilitating mitochondrial autophagy and healthy mitochondrial production. Furthermore, phenolics induce autophagy by directly inhibiting the mTOR/ULK1 pathway because mTORC1 is activated and phosphorylates ULK1 and ATG13 to inhibit autophagosome formation under nutrient-rich and energy-sufficient conditions. The phenolic-mediated mTOR signaling pathway also involves indirect regulation of SIRT1 and AMPK. Phenolics increase mitochondrial autophagy by upregulating p53 deacetylation to activate SIRT1, which downregulates phosphorylation of AKT and mTOR in PI3K/AKT/mTOR and upregulates the expression of ATG5, ATG7, LC3, and ATG5–ATG12 complexes. Under nutrient depletion, AMPK activation induces the phosphorylation of the Raptor portion making up the mTORC1 complex, thereby inhibiting mTORC1 activity and lifting mitochondrial autophagy restriction. However, mitochondrial autophagy is a double-edged sword, and excessive autophagy is another form of programmed cell death that prevents cell arousal and regeneration, as seen in aging cells (Murley et al., 2025) and in diseases such as myocardial ischemia/reperfusion (Chen et al., 2021), arthritis (Sarkar et al., 2022), neurodegeneration (Pluta et al., 2022), and cancer (Kong et al., 2022). At this time, phenolics such as curcumin, resveratrol, and EGCG reduce mitochondrial autophagy by inhibiting PINK1/Parkin, activating PI3K/AKT/mTOR, or inhibiting JNK/p38 MAPK signaling pathways. In addition to cell type, the biphasic effects of phenolics also depend on dosage, duration of exposure, and the cellular environment. With low to moderate doses, short-term treatment, and low basal oxidative stress levels, phenolics tend to induce mitochondrial autophagy. The good tolerance of phenolics, which position mitochondria similarly to calorie restriction, is another major reason for their anti-aging.

#### 4.1.5 Maintenance of intestinal function

Crosstalk between the gut cell mitochondria–gut microbiota axis has been reported by Michaudel and Sokol (2020) and Kulkarni and Gaikwad (2025). Gut microbes can provide a source of energy for gut cells and enhance mitochondrial function by participating in nutrient metabolism, modulating host cell-signaling pathways, and reducing oxidative stress levels. Mitochondria provide energy for normal physiological functions of gut cells and participate in the activation and regulation of signaling pathways, including maintaining gut mucosal integrity and effectively monitoring gut microbes. Conversely, when gut flora is dysbiotic or mitochondrial function is impaired, it may lead to dysregulation of the gut immune system and alteration of the composition of the gut microbial community, causing inflammatory infections and oxidative stress. Interestingly, there are also interactions and influences between phenolics and gut microbes. On the one hand, gut microbes can biotransform phenolics into small-molecule phenolic acids through hydrolysis, cleavage, and demethylation so that they are exposed and absorbed to a greater extent, thus having better effects on the mitochondria of immune cells such as the gut mucosal cells, epithelial cells, lymphocytes, and macrophages in the way described above (Li et al., 2024b). On the other hand, phenolics are also potential prebiotics for the gut and can remodel gut microbes by differentially adjusting them to inhibit pathogen invasion (e.g., lowering the ratio of *Firmicutes*/*Bacteroidetes*, which is related to metabolic disorders, and upregulating the percentage of *Akkermansia muciniphila* and *Roseburia*) (Gowd et al., 2019). After synthesizing results from 44 studies, Perez-Jimenez et al. (2023) shockingly found that phenolics can regulate the circadian rhythm through the gut in association with the distal brain, while sleep itself emerges as the easiest mechanism for mitochondrial restoration.

#### 4.1.6 Other roles

In addition to mitochondria-specific effects, phenolics can also improve mitochondria and oxidative damage by taking cellular-level initiatives such as resisting apoptosis, revitalizing stem cells, and removing senescent cells. For instance, quercetin significantly ameliorated mitochondrial pathway-mediated apoptosis by modulating BAX and BCL-2 expression and inhibiting caspase-3 activation in an acrylamide-induced mouse model of liver injury (Li et al., 2024c). The study of hawthorn polyphenol extract by Liu et al. (2019) also showed that it could regulate the p53 mitochondrial pathway (upregulation of BCL-2 expression, downregulation of BAX expression, and attenuation of caspase-3/9 activation) to inhibit UV-induced skin oxidative stress damage. Other studies included that ferulic acid improved mitochondria, thereby promoting AMPK expression and reducing apoptosis to attenuate hypoxia/reoxygenation damage in renal tubular epithelial cells (Chen et al., 2022), and allicin inhibited osteoblast apoptosis and steroid-induced necrosis of the femoral head by regulating the aberrant expression of caspase-3/9, BAX, and BCL-2 through the activation of the PI3K/AKT pathway (Zhan et al., 2020). Additionally, An et al. (2022) considered neurological disorders as research subjects and summarized the induced regenerative effects of polyphenols, phenolic acids, and flavonoids on neural and mesenchymal stem cells, providing a therapeutic basis for subsequent phenolic enhancement of osteoblastic and gut stem cell activity. Meanwhile, phenolics represented by quercetin (Millar et al., 2025), procyanidin C1 (Shao et al., 2024), 8-paradol (Wang et al., 2023), and *Olea europaea* leaf phenolic extract (Ercelik et al., 2023) could reduce lower back pain, improve bone density, inhibit lung fibrosis, and treat cancer by exacerbating the mitochondrial autophagy or mitochondrial apoptosis pathway to remove senescent cells.

Phenolics are emerging as a new force in the discipline of food-based anti-aging by simultaneously regulating mitochondrial oxidative stress through different biochemical processes. These compelling efficacy profiles have become the foundation for promoting phenolics in health science.

### 4.2 Delivery strategies of phenolics in managing mitochondrial aging

**FIGURE 5:**
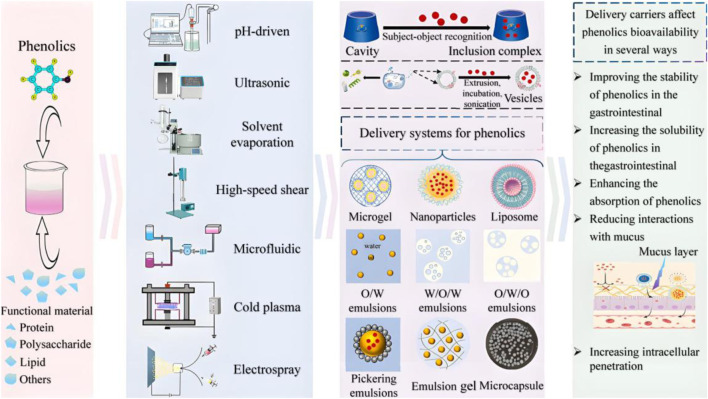
Technology roadmap for the construction of delivery systems and potential ways for delivery systems to improve the bioavailability of phenolics (modified from Wang W. et al., 2025).

Though food is the primary route for people to consume phenolics, their properties, such as sensitivity to light, heat, and oxygen; low oral utilization (less than 1%); limited absorption and metabolism; and rapid dissipation, make it necessary to augment the regular diet with small quantities of nutrient-dense supplements to reduce the dietary burden and achieve clinically effective concentrations. Oral delivery based on human compliance is the preferred option, but transporting phenolics from the harsh environment of the gastrointestinal tract to the systemic circulation presents many challenges. At present, phenolics are primarily administered in humans as traditional capsules and tablets taken with water, but problems such as limited solubility, unstable oral bioavailability, and uneven distribution in body fluids remain unresolved. The transformative strategy of preparing phenolics into nanomaterials using advanced carriers has gained attention for the ability to increase their solubility, enhance their digestive stability, bring them closer to the scale of biomolecules (<500 nm) to penetrate biological barriers, prolong the diffusion time through sustained release, and specifically target organs (Ejazi et al., 2023). Lu Y. et al. (2025), Xiang et al. (2024), Wang W. et al. (2025), and Liu K. et al. (2025) outlined this, which mainly included nanoemulsions, nanoparticles, liposomes, inclusions, nanofibers, nanomicelles, nanosheets, gels, and nanovesicles, as shown in their morphology and preparation methods in Figure 5. Physical field-driven self-assembly and emerging technologies are being developed integrally. There is a broad range of carrier materials, including natural substances, synthetic substances, and metals/oxides. Many other nanosystems, such as plant-derived nanoparticles and virus-like nanoparticles, are also being investigated, and details on their types, properties, and delivery applications are available in the recent review coverage. They can increase bioavailability by several- to more than a dozen-fold compared with the oral bioavailability of free phenolics. It should be emphasized that supplement formulations are quite different from oral administration as the selection of supplement carriers requires careful consideration of long-term edibility and market prices in the food category. Therefore, substances such as proteins and lipids may be preferable, as reported by Tang et al. (2023) and Tang (2021). Oral nanodelivery of phenolics is progressively advancing beyond cellular and animal studies to human applications, such as the clinical translation of curcumin-based nanopreparations (Jacob et al., 2024) and the evaluation of a composite polyphenol nanoparticle drink in individuals with insomnia, as documented in patent CN116889248A. Every effort and dedication have propelled phenolics from laboratory discovery to widespread use in alleviating aging-related symptoms and diseases. The core limitation of this application lies in dose inefficacy as most compounds are not effectively utilized, and the actual active agent may differ from the original substance. Future research must shift from pursuing prototype compounds to revealing the network effects of their individualized metabolites.

## 5 Mitochondrial aging intervention based on long-term moderate exercise

As we all know, exercise is inherently a stressor for the body as it increases ROS production and decreases oxygen supply to organs, but it is also essential for overall wellbeing of humans, supporting both physical and mental health. In the extensive discussion on the public topic of exercise, relevant experts have confirmed that various exercise modalities, such as aerobic, anaerobic, resistance, and balance, and their combinations have anti-aging potential through data support and factual basis provided by randomized controlled trials and statistical and meta-analysis, which can increase metabolic energy of the body and improve skin aging and common ailments, such as obesity, muscle mass, diabetes, neurodegenerative diseases, cardiovascular disease, and tumors, and reduce all-cause mortality (Amar et al., 2024; Jia et al., 2023; Lu X. et al., 2025). In response, the World Health Organization published physical activity guidelines and strongly recommended that all adults, including people with chronic diseases and syndromes, should perform at least 150 min–300 min of moderate-intensity aerobic exercise (brisk walking, jogging, bicycling, swimming, housework, etc.), 75 min–150 min of vigorous-intensity aerobic exercise (fast running, high-intensity interval training, etc.), or an equivalent combination of the two (moderate-to-vigorous physical activity), along with at least 2 days of moderate-intensity strength training (push-ups, dumbbells, etc.) per week with strict limits on sedentary time (Bull et al., 2020).

**FIGURE 6:**
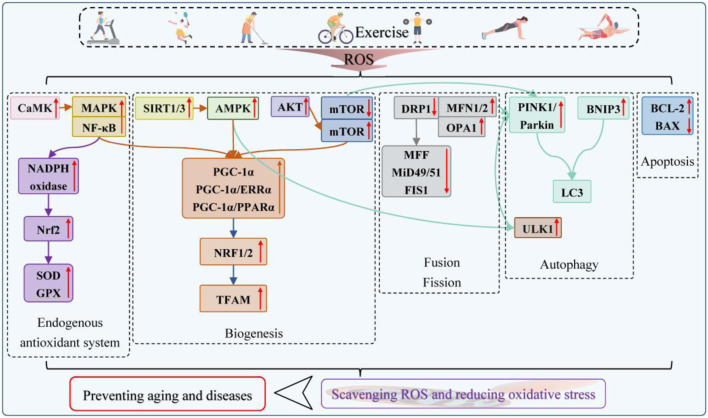
*In vivo* pathways for the elimination of oxidative stress by exercise.

The rationale for the necessity of exercise is that it significantly modifies mitochondrial morphology and function (Figure 6). Exercise allows the body to acquiesce to ROS as a “hormetic” signal and make active adaptive changes, which can effectively prevent mitochondrial oxidative damage and prolong the tolerable oxidative level, one principle of which is the upregulation of antioxidant enzymes and the reduction of oxidants. Hansen et al. (2022) found that in sedentary men who underwent 50-min high-intensity aerobic interval training three times per week for 8 weeks the expression of SOD and GPX increased in the thigh muscles endothelial cells by 2.4 times and 2.3 times, respectively. Enhanced antioxidant activity was driven by NADPH oxidase activation of Nrf2, both of which promoted aerobic fitness of skeletal muscle mitochondria and eliminated the pro-fatigue effects of harmful substances (Galvan-Alvarez et al., 2023).

As opposed to muscle non-use, exercise enhances mitochondrial dynamics and lysosomal expression to improve the mitochondrial quality and remove defective mitochondria and accumulated cellular debris. Mitochondrial proliferation signals were observed in human skeletal muscle under endurance and resistance protocols. The controlled production of ROS and their induced MAPK or NF-κB to regulate PGC-1α/TFAM expression at the upstream was initially recognized (Dimauro et al., 2020). Recent research on this topic by Ravindran and Gustafsson (2025), Yuan et al. (2025), and Fan, et al. (2024) revealed that PGC-1α activation was also associated with SIRT1/3/AMPK and CaMK/MAPK. The PGC-1α/ERRα and PGC-1α/PPARα axes act as bridges connecting AMPK and NRF1/2 to promote mitochondrial biogenesis and increase mitochondrial density in muscle training, obesity, sarcopenia, and environmental exposures by inducing TFAM transcription, which is primarily targeted to aerobic and endurance exercise. On the other hand, muscle contraction (high-intensity interval training), certain acute exercise, and regular aerobic exercise are prescriptions for heart failure that increase mitochondrial biogenesis by activating the CaMK/MAPK to restore Ca^2+^ cycling efficiency. When mechanical loads are introduced to enhance muscle fiber hypertrophy, mitochondrial biogenesis is switched on by the AKT/mTOR or direct mTOR activation pathway to meet the high requirements of amino acid uptake rate, cytoplasmic myonuclei number, and percentage (Yoon, 2017).

Yu et al. (2023) found that aerobic exercise and high-intensity interval training could downregulate DRP1 and its recruited MFF, MiD49/51, and FIS1 and upregulate MFN1/2 and OPA1, suggesting antioxidant therapeutic strategies for fatty liver and hypertension that target mitochondrial fusion and fission. Long-term aerobic exercise such as running and swimming, resistance exercise, and free exercise are all effective in restoring mitochondrial autophagy affected by the oxidative stress of aging or disease. The consensual account is that exercise directly mediates the PINK1/Parkin or BNIP3 pathway and activates AMPK/ULK1 to phosphorylate Parkin and re-localize it at the mitochondrial outer membrane to drive contact with LC3 (Lin et al., 2025). Andani et al. (2024) further revealed that high-intensity interval training and aerobic exercise improved the interaction of MFN2 with Parkin as another way to increase mitochondrial involvement in autophagy. Similar to nutritional deprivation, AMPK activation inhibits mTORC1 and promotes TFEB/TFE3 nuclear translocation and lysosomal biogenesis during exercise, and mitochondrial ROS play a crucial role in enhancing lysosomal calcium release (Moradi et al., 2024).

Additionally, some unexpected benefits of exercise on mitochondria have also been reported. For example, swimming upregulated BCL-2 and downregulated BAX expression, and after 8 weeks, cardiomyocyte apoptosis was reversed (Ma et al., 2025); a systematic review and meta-analysis led by Min et al. (2024) showed that exercise significantly increased gut microbial diversity in adults and the ratio of *Firmicutes*/*Bacteroidetes* associated with enteritis and Alzheimer’s disease; exercise also robustly regulates the skeletal muscle clock and resets the molecular circadian clock, thereby ameliorating the negative effects of disturbed sleep patterns (Gabriel and Zierath, 2019); muscle biopsies from subjects after intermittent completion of 15 sets of 20 s sprint rides in a randomized, double-blind, and crossover trial showed that high-intensity exercise effectively cleared skeletal muscle senescent cells within 3 h for more than 24 h and promoted muscle regeneration through post-inflammatory tissue repair processes (Jean et al., 2024).

While the efficacy determination depends on age, gender, environment, and nutrition, all benefits arise from adherence and moderation. Acute exercise serves as a stress test for mitochondria, evaluating their immediate energy supply capacity. A series of response signals (elevated Ca^2+^ concentration, massive ROS production, increased ADP/ATP, and decreased NAD^+^/NADH) lay the groundwork for subsequent chronic exercise adaptation. However, acute exercise may cause bodily damage when the stress endured is insufficient to counteract oxidative stress. Chronic exercise is essentially upgrading cells. It is based on signals triggered by acute exercise to enhance mitochondrial resilience, ultimately creating healthier energy powerhouses (Kankaanpaeae et al., 2025; Sharma and Mehdi, 2023).

## 6 Phenolics in combination with exercise: a double play for epigenetic health and youth

It is known from studies in mouse models and observations in human cells that reversing mitochondrial damage could restore the original ability to control sugar in diabetics (Walker et al., 2025). Taken together, phenolics and exercise weave a multifaceted network to govern mitochondria based on cross-presence and mutually causal cascade pathways. Phenolics and exercise both are aimed at promoting normal mitochondrial stress and activating their own cardio-protective and anti-aging mechanisms. The high correlation of goals and pathways exhibited by the two triggered the exploration of their combined use.

Through literature review, it has been observed that their intersection was mostly focused on phenolics to improve exercise performance. As Liu Y. et al. (2025) reported, salidroside could inhibit muscle lactate vesicle formation to attenuate overtraining-induced liver fibrosis. A randomized, single-blind, placebo-controlled, and crossover trial demonstrated that chicory enhanced aerobic exercise and subsequent recovery in adult students by facilitating lactate oxidation (Mao et al., 2025). Oral garlic extract supplements enhanced glycogen replenishment and protein synthesis after exercise (Cheng et al., 2024). The existing valuable information is exemplified by the interaction of resveratrol with 6 weeks of high-intensity interval swimming training to reduce oxidative stress in the frontal mitochondria of aged rats (Mehrabi et al., 2025), adjustment to a plant-based diet and taking polyphenol nutrient supplements twice daily for 8 weeks, which reduced participants’ biological age by an average of 4.6 years (Fitzgerald et al., 2023), and a 6-month randomized intervention combining aerobic exercise and phenolic-rich, which reduced liver fat and prevented diabetes by improving the diversity and stability of key gut microbes (Zhang et al., 2022), highlighting the synergistic anti-aging benefits of phenolics and exercise. Despite the limitations and lack of expanded human studies, based on the support of 18,738 middle-aged or young adults who displayed aging attenuation with vitamin D supplementation combined with physical activity (Liu C. et al., 2024) and the results of a cross-sectional study of 41 middle-aged and older adults by Fiorito et al. (2025) that long-term caloric restriction complemented by endurance exercise could combat aging on all fronts (especially in the gut), the future of phenolic supplements plus physical activity is predictable. This explains well that their long-term collaboration not only overcomes the aging inevitability driven by tissue damage and age but may also be a powerful tool for eradicating chronic disease, which moves closer to the grand vision of carbon neutrality and peak carbon and promises to disrupt conventional therapeutic approaches. Choosing a phenolic supplement window that is easy to stick with according to the lifestyle and work schedule, trying to exercise during this window, and starting with low-dose supplementation and lighter exercise intensity may synchronize a phenolic diet and exercise with the biological clock for optimal health.

## 7 A revisit of the potential problems and solutions

Based on the global situation and future trends of the industry, phenolic supplements combined with exercise provide a new perspective on health for all of mankind. However, in the journey toward popularization, implementation of standardization and concretization needs to consider a variety of factors, not just a simple combination.

The details are as follows:

1. The multi-potency, minimal side effects, and nanosizing of food-derived phenolics have enabled them to overcome barriers to scientific innovation and commercial translation. The unknown risks from bioaccumulation of nano-additives in the body, environmental contamination with nanoparticles during manufacturing processes, the differences in sensitivity acceptance across populations, and the still imperfect regulatory policies in different countries have led to some controversy regarding the safety of nano-enabled phenolic applications. Hence, accelerating the establishment of a harmonized standard framework in the global arena, strengthening long-term consumption studies of phenolic nanosupplements, exploring phenolic synergism, and developing delivery systems for enhanced mitochondrial targets will help ensure their efficient and safe use in the market and provide consumers with a reliable anti-aging guarantee.
2. Because of the biphasic effects and tissue specificity of phenolics and exercise, high doses of phenolics and overactivity are instead detrimental to the body due to oxidation. However, there are no clear protocols or biomarkers to differentiate between moderate and excessive application and predict health effects. Therefore, obtaining clinical differences in redox balance thresholds between individuals for the combination of the two to determine their safe dosages, durations, and combinations is imperative.
3. In the process of fleshing out anti-aging research on phenolic supplements combined with exercise, using genomic techniques such as gene, transcription, protein, and single-cell metabolism to focus on the integration of mitochondrial function and phenolic and exercise anti-aging mechanisms, advanced techniques such as artificial intelligence and machine learning are more fully applied to elucidate the understanding of the impact of their combination on mitochondrial quality control.
4. Leveraging artificial intelligence models to quantify ROS, phenolic intake, physical activity, gut flora, and gene expression through image recognition, motion analysis, and intelligent detection and developing cost-effective instrumentation and user-friendly platforms to design precise anti-aging programs for individuals and dynamically evaluate the therapeutic effect will be the future trend in health management.
5. For decades, interventions that solely rely on changing the food environment have improved the dietary choices of people to some extent, but they have not fundamentally reformed the food supply system. Encouraging individuals to change their dietary and exercise habits is challenging. This requires the participation of policymakers, experts, scholars, and all sectors of society to promote the formation of public anti-aging awareness and create a cultural atmosphere of health.

## 8 Conclusion

The question of aging nature is answered based on mitochondrial properties and the influence of ROS. The well-documented data also explain this and provide basic information on mitochondrial dysfunction and disease during aging. By examining the causes and processes underlying aging, scientific research has revealed the complex interplay of phenolics and exercise in regulating mitochondrial oxidative stress. This includes direct actions, such as scavenging ROS, upregulating antioxidant enzymes, and improving mitochondrial dynamics, along with indirect cellular-level effects, highlighting their anti-aging potential. Owing to their interplay, nanophenolics with enhanced bioavailability and pharmacokinetics, in conjunction with exercise for enhanced anti-aging effects, have been proposed and considered with significant expectations, representing an integration of modern biology and traditional plant wisdom. The strategy is highly adaptable, but widespread application has been constrained by unresolved factors, including newly introduced policies (major agencies such as China’s State Administration for Market Regulation and the U.S. Food and Drug Administration strictly prohibit the addition of pharmaceutical ingredients to foods, and claims of “anti-aging” effects for food or general health supplements face extremely stringent regulatory restrictions), pending security reviews (the unknown risks associated with combining phenolics with exercise, coupled with the ambiguity surrounding their optimal dosage), and critical scientific challenges requiring resolution. It is anticipated that the combined regimen of phenolic supplements and exercise can inform healthy choices and healthcare in the near future, as standardized and clearly defined protocols are gradually refined.

## Glossary

ROS: Reactive oxygen species
NADPH: Nicotinamide adenine dinucleotide phosphate hydrogen
SOD: Superoxide dismutase
CAT: Catalase
GPX: Glutathione peroxidase
Nrf2: Nuclear factor-erythroid 2-related factor-2
PGC-1α: Peroxisome proliferator-activated receptor γ coactivator alpha
TFAM: Mitochondrial transcription factor A
AMPK: Adenosine 5’-monophosphate-activated protein kinase
SIRT1: NAD^+^-dependent deacetylase sirtuin-1
MFN1/2: Mitofusin 1/2
OPA1: Optic atrophy 1
DRP1: Dynamin-related protein 1
PINK1: PTEN-induced putative kinase 1
Parkin: Parkinson’s disease
BNIP3: BCL2 interacting protein 3
NIX: NIP3-like protein X
LC3: Microtubule-associated protein 1 light chain 3
LIR: LC3 interaction region
ATP: Adenosine triphosphate
**NAD** ^**+**^: Nicotinamide adenine dinucleotide
Keap1: Kelch-like ECH associated protein 1
NQO1: NAD(P)H quinone dehydrogenase 1
HO-1: Heme oxygenase 1
NADH: Nicotinamide adenine dinucleotide
COX: Cyclooxygenase
LOX: Lipoxygenase
NF-κB: Nuclear factor kappa-B
MAPK: Mitogen-activated protein kinase
PI3K: Phosphoinositide 3-kinase
AKT: Protein kinase B
NAMPT: Nicotinamide phosphoribosyl transferase
NMNAT: Nicotinamide mononucleotide adenylyl transferase
FoxO3: Forkhead box O3
NRF1/2: Nuclear respiratory factor 1/2
EGCG: Epigallocatechin gallate
FIS1: Fission 1
OPTN: Optineurin
NDP52: Nuclear dot protein 52
VDAC1: Voltage-dependent anion channel 1
SIRT3: NAD^+^-dependent deacetylase sirtuin-3
FoxO1: Forkhead box O1
CaMKKβ: Ca^2+^/calmodulin-dependent protein kinase kinase beta
LKB1: Liver kinase B1
ULK1: UNC-51-like kinase 1
mTOR: Mammalian target of rapamycin
ATG13: Autophagy related gene 13
ATG5: Autophagy related gene 5
ATG7: Autophagy related gene 7
JNK: c-Jun N-terminal kinase
BAX: BCL-2-associated X protein
BCL-2: B-cell lymphoma-2
ERRα: Estrogen-related receptor alpha
PPARα: Peroxisome proliferator-activated receptor alpha
CaMK: Calmodulin kinase
MFF: Mitochondrial fission factor
MiD49/51: Mitochondrial dynamics proteins of 49 kDa/51 kDa
TFEB: Transcription factor EB
TFE3: Transcription factor E3

## References

[B1] Abu Shelbayeh O. Arroum T. Morris S. Busch K. B. (2023). PGC-1α is a master regulator of mitochondrial lifecycle and ROS stress response. Antioxidants 12 (5), 1075. 10.3390/antiox12051075 37237941 PMC10215733

[B2] Amar D. Gay N. R. Jean-Beltran P. M. Bae D. Dasari S. Dennis C. (2024). Temporal dynamics of the multi-omic response to endurance exercise training. Nature 629 (8010), 174–183. 10.1038/s41586-023-06877-w 38693412 PMC11062907

[B3] Amorim J. A. Coppotelli G. Rolo A. P. Palmeira C. M. Ross J. M. Sinclair D. A. (2022). Mitochondrial and metabolic dysfunction in ageing and age-related diseases. Nat. Rev. Endocrinol. 18 (4), 243–258. 10.1038/s41574-021-00626-7 35145250 PMC9059418

[B4] An J. Chen B. Tian D. Guo Y. Yan Y. Yang H. (2022). Regulation of neurogenesis and neuronal differentiation by natural compounds. Curr. Stem Cell Res. Ther. 17 (8), 756–771. 10.2174/1574888x16666210907141447 34493197

[B5] Andani F. M. Talebi-Garakani E. Ashabi G. Ganbarirad M. Hashemnia M. Sharifi M. (2024). Exercise-activated hepatic autophagy combined with silymarin is associated with suppression of apoptosis in rats subjected to dexamethasone induced-fatty liver damage. Mol. Biol. Rep. 51 (1), 928. 10.1007/s11033-024-09844-4 39172304

[B6] Argentieri M. A. Amin N. Nevado-Holgado A. J. Sproviero W. Collister J. A. Keestra S. M. (2025). Integrating the environmental and genetic architectures of aging and mortality. Nat. Med. 31, 1016–1025. 10.1038/s41591-024-03483-9 39972219 PMC11922759

[B7] Arruda H. S. Neri-Numa I. A. Kido L. A. Marostica Junior M. R. Pastore G. M. (2020). Recent advances and possibilities for the use of plant phenolic compounds to manage ageing-related diseases. J. Funct. Foods 75, 104203. 10.1016/j.jff.2020.104203

[B8] Avila-Roman J. Soliz-Rueda J. R. Isabel Bravo F. Aragones G. Suarez M. Arola-Arnal A. (2021). Phenolic compounds and biological rhythms: who takes the lead? Trends Food Sci. Technol. 113, 77–85. 10.1016/j.tifs.2021.04.050

[B9] Beard J. R. Officer A. de Carvalho I. A. Sadana R. Pot A. M. Michel J. P. (2016). The World report on ageing and health: a policy framework for healthy ageing. Lancet 387 (10033), 2145–2154. 10.1016/s0140-6736(15)00516-4 26520231 PMC4848186

[B10] Behr L. C. Simm A. Kluttig A. Grosskopf A. G. (2023). 60 years of healthy aging: on definitions, biomarkers, scores and challenges. Ageing Res. Rev. 88, 101934. 10.1016/j.arr.2023.101934 37059401

[B11] Boengler K. Kosiol M. Mayr M. Schulz R. Rohrbach S. (2017). Mitochondria and ageing: role in heart, skeletal muscle and adipose tissue. J. Cachexia Sarcopenia Muscle 8 (3), 349–369. 10.1002/jcsm.12178 28432755 PMC5476857

[B12] Bull F. C. Al-Ansari S. S. Biddle S. Borodulin K. Buman M. P. Cardon G. (2020). World Health Organization 2020 guidelines on physical activity and sedentary behaviour. Br. J. Sports Med. 54 (24), 1451–1462. 10.1136/bjsports-2020-102955 33239350 PMC7719906

[B13] Cai Y. Song W. Li J. Jing Y. Liang C. Zhang L. (2022). The landscape of aging. Sci. China-Life Sci. 65 (12), 2354–2454. 10.1007/s11427-022-2161-3 36066811 PMC9446657

[B14] Calubag M. F. Robbins P. D. Lamming D. W. (2024). A nutrigeroscience approach: dietary macronutrients and cellular senescence. Cell Metab. 36 (9), 1914–1944. 10.1016/j.cmet.2024.07.025 39178854 PMC11386599

[B15] Castro L. d. S. Bracht L. Peralta R. M. Marostica H. V. P. Comar J. F. Sa-Nakanishi A. B. d. (2023). Free radical quenching in liver mitochondria by selected antioxidants abundant in foods and supplements. Food Biosci. 54, 102926. 10.1016/j.fbio.2023.102926

[B16] Chen Y. Chen J. Sun X. Shi X. Wang L. Huang L. (2018). Evaluation of the neuroprotective effect of EGCG: a potential mechanism of mitochondrial dysfunction and mitochondrial dynamics after subarachnoid hemorrhage. Food Funct. 9 (12), 6349–6359. 10.1039/c8fo01497c 30452052

[B17] Chen X. Xie Q. Zhu Y. Xu J. Lin G. Liu S. (2021). Cardio-protective effect of tetrahydrocurcumin, the primary hydrogenated metabolite of curcumin *in vivo* and *in vitro:* induction of apoptosis and autophagy *via* PI3K/AKT/mTOR pathways. Eur. J. Pharmacol. 911, 174495. 10.1016/j.ejphar.2021.174495 34555398

[B18] Chen T. Niu L. Wang L. Zhou Q. Zhao X. Lai S. (2022). Ferulic acid protects renal tubular epithelial cells against anoxia/reoxygenation injury mediated by AMPKα1. Free Radic. Res. 56 (2), 173–184. 10.1080/10715762.2022.2062339 35382666

[B19] Chen W. Zhao H. Li Y. (2023). Mitochondrial dynamics in health and disease: mechanisms and potential targets. Signal Transduct. Target. Ther. 8 (1), 333. 10.1038/s41392-023-01547-9 37669960 PMC10480456

[B20] Chen J. Zhou Z. Wu N. Li J. Xi N. Xu M. (2024). Chlorogenic acid attenuates deoxynivalenol-induced apoptosis and pyroptosis in human keratinocytes *via* activating Nrf2/HO-1 and inhibiting MAPK/NF-κB/NLRP3 pathways. Biomed. Pharmacother. 170, 116003. 10.1016/j.biopha.2023.116003 38091639

[B21] Chen S. Li Q. Shi H. Li F. Duan Y. Guo Q. (2024). New insights into the role of mitochondrial dynamics in oxidative stress-induced diseases. Biomed. Pharmacother. 178, 117084. 10.1016/j.biopha.2024.117084 39088967

[B22] Cheng I. S. Tsao J. P. Bernard J. R. Tsai T. W. Chang C. C. Liao S. F. (2024). Oral post-exercise garlic extract supplementation enhances glycogen replenishment but does not up-regulate mitochondria biogenesis mRNA expression in human-exercised skeletal muscle. J. Int. Soc. Sports Nutr. 21 (1), 2336095. 10.1080/15502783.2024.2336095 38576169 PMC11000618

[B23] Chini C. C. S. Cordeiro H. S. Tran N. L. K. Chini E. N. (2024). NAD metabolism: role in senescence regulation and aging. Aging Cell 23 (1), e13920. 10.1111/acel.13920 37424179 PMC10776128

[B24] Davinelli S. De Stefani D. De Vivo I. Scapagnini G. (2020). Polyphenols as caloric restriction mimetics regulating mitochondrial biogenesis and mitophagy. Trends Endocrinol. Metabolism 31, 536–550. 10.1016/j.tem.2020.02.011 32521237

[B25] Dimauro I. Paronetto M. P. Caporossi D. (2020). Exercise, redox homeostasis and the epigenetic landscape. Redox Biol. 35, 101477. 10.1016/j.redox.2020.101477 32127290 PMC7284912

[B26] Du S. Zhu X. Zhou N. Zheng W. Zhou W. Li X. (2022). Curcumin alleviates hepatic steatosis by improving mitochondrial function in postnatal overfed rats and fatty L02 cells through the SIRT3 pathway. Food Funct. 13 (4), 2155–2171. 10.1039/d1fo03752h 35113098

[B27] Dudylina A. L. Ivanova M. V. Shumaev K. B. Ruuge E. K. (2019). Superoxide formation in cardiac mitochondria and effect of phenolic antioxidants. Cell Biochem. Biophy. 77 (1), 99–107. 10.1007/s12013-018-0857-2 30218405

[B28] Ejazi S. A. Louisthelmy R. Maisel K. (2023). Mechanisms of nanoparticle transport across intestinal tissue: an oral delivery perspective. Acs Nano 17 (14), 13044–13061. 10.1021/acsnano.3c02403 37410891

[B29] Ercelik M. Tekin C. Tezcan G. Aksoy S. A. Bekar A. Kocaeli H. (2023). *Olea europaea* leaf phenolics oleuropein, hydroxytyrosol, tyrosol, and rutin induce apoptosis and additionally affect temozolomide against glioblastoma: in particular, oleuropein inhibits spheroid growth by attenuating stem-like cell phenotype. Life-Basel 13 (2), 470. 10.3390/life13020470 36836827 PMC9964321

[B30] Fan D. Pan K. Guo J. Liu Z. Zhang C. Zhang J. (2024). Exercise ameliorates fine particulate matter-induced metabolic damage through the SIRT1/AMPKα/PGC1-α/NRF1 signaling pathway. Environ. Res. 245, 117973. 10.1016/j.envres.2023.117973 38145729

[B31] Fang Y. Xia J. Lian Y. Zhang M. Kang Y. Zhao Z. (2023). The burden of cardiovascular disease attributable to dietary risk factors in the provinces of China, 2002-2018: a nationwide population-based study. Lancet Regional Health. West. Pac. 37, 100784. 10.1016/j.lanwpc.2023.100784 37693878 PMC10485670

[B32] Favari C. de Alvarenga J. F. R. Sanchez-Martinez L. Tosi N. Mignogna C. Cremonini E. (2024). Factors driving the inter-individual variability in the metabolism and bioavailability of (poly)phenolic metabolites: a systematic review of human studies. Redox Biol. 71, 103095. 10.1016/j.redox.2024.103095 38428187 PMC10912651

[B33] Felix J. Martinez de Toda I. Diaz-Del Cerro E. Gonzalez-Sanchez M. De la Fuente M. (2024). Frailty and biological age. Which best describes our aging and longevity? Mol. Aspects Med. 98, 101291. 10.1016/j.mam.2024.101291 38954948

[B34] Fiorito G. Tosti V. Polidoro S. Bertozzi B. Veronese N. Cava E. (2025). Multi-omic analysis of biological aging biomarkers in long-term calorie restriction and endurance exercise practitioners: a cross-sectional study. Aging Cell 24 (4), e14442. 10.1111/acel.14442 39692728 PMC11984672

[B35] Fitzgerald K. N. Campbell T. Makarem S. Hodges R. (2023). Potential reversal of biological age in women following an 8-week methylation-supportive diet and lifestyle program: a case series. Aging-Us 15 (6), 1833–1839. 10.18632/aging.204602 36947707 PMC10085584

[B36] Gabriel B. M. Zierath J. R. (2019). Circadian rhythms and exercise - re-setting the clock in metabolic disease. Nat. Rev. Endocrinol. 15 (4), 197–206. 10.1038/s41574-018-0150-x 30655625

[B37] Gai Z. Hu S. Gong G. Zhao J. (2023). Recent advances in understanding dietary polyphenols protecting against hypertension. Trends Food Sci. Technol. 138, 685–696. 10.1016/j.tifs.2023.07.008

[B38] Galvan-Alvarez V. Gallego-Selles A. Martinez-Canton M. Garcia-Gonzalez E. Gelabert-Rebato M. Ponce-Gonzalez J. G. (2023). Antioxidant enzymes and Nrf2/Keap1 in human skeletal muscle: influence of age, sex, adiposity and aerobic fitness. Free Radic. Biol. Med. 209, 282–291. 10.1016/j.freeradbiomed.2023.10.393 37858747

[B39] Gherardi G. Weiser A. Bermont F. Migliavacca E. Brinon B. Jacot G. E. (2025). Mitochondrial calcium uptake declines during aging and is directly activated by oleuropein to boost energy metabolism and skeletal muscle performance. Cell Metab. 37 (2), 477–495.e11. 10.1016/j.cmet.2024.10.021 39603237

[B40] Gowd V. Karim N. Shishir M. R. I. Xie L. Chen W. (2019). Dietary polyphenols to combat the metabolic diseases *via* altering gut microbiota. Trends Food Sci. Technol. 93, 81–93. 10.1016/j.tifs.2019.09.005

[B41] Gu Y. Han J. (2023). Autophagy and polyphenol intervention strategy in aging. Trends Food Sci. Technol. 132, 1–10. 10.1016/j.tifs.2022.12.013

[B42] Guo Z. Liu H. Zhao D. Wang X. Zang Z. Wang G. (2025). Piperine ameliorates diabetic mellitus erectile dysfunction by reducing oxidative stress and apoptosis through the PI3K/AKT/NRF2 signaling pathway. Food Biosci. 66, 106326. 10.1016/j.fbio.2025.106326

[B43] Han L. Ho C. T. Lu M. (2025). Regulatory role of bioactive compounds from natural spices on mitochondrial function. J. Agric. Food Chem. 73 (10), 5711–5723. 10.1021/acs.jafc.4c12341 40019340

[B44] Hansen C. Moller S. Ehlers T. Wickham K. A. Bangsbo J. Gliemann L. (2022). Redox balance in human skeletal muscle-derived endothelial cells-effect of exercise training. Free Radic. Biol. Med. 179, 144–155. 10.1016/j.freeradbiomed.2021.12.265 34954023

[B45] Harman D. (1956). Aging: a theory based on free radical and radiation chemistry. J. Gerontol. 11 (3), 298–300. 10.1093/geronj/11.3.298 13332224

[B46] He Z. Zhang J. Xu Y. Fine E. J. Suomivuori C. M. Dror R. O. (2025). Structure of mitochondrial pyruvate carrier and its inhibition mechanism. Nature 641, 250–257. 10.1038/s41586-025-08667-y 40044865 PMC12043432

[B47] Hong Y. Boiti A. Vallone D. Foulkes N. S. (2024). Reactive oxygen species signaling and oxidative stress: transcriptional regulation and evolution. Antioxidants 13 (3), 312. 10.3390/antiox13030312 38539845 PMC10967436

[B48] Hussain T. Tan B. Yin Y. Blachier F. Tossou M. C. B. Rahu N. (2016). Oxidative stress and inflammation: what polyphenols can do for us? Oxidative Med. Cell. Longev. 2016, 7432797. 10.1155/2016/7432797 27738491 PMC5055983

[B49] Iorio R. Petricca S. Mattei V. Delle Monache S. (2024). Horizontal mitochondrial transfer as a novel bioenergetic tool for mesenchymal stromal/stem cells: molecular mechanisms and therapeutic potential in a variety of diseases. J. Transl. Med. 22 (1), 491. 10.1186/s12967-024-05047-4 38790026 PMC11127344

[B50] Jacob S. Kather F. S. Morsy M. A. Boddu S. H. S. Attimarad M. Shah J. (2024). Advances in nanocarrier systems for overcoming formulation challenges of curcumin: current insights. Nanomaterials 14 (8), 672. 10.3390/nano14080672 38668166 PMC11054677

[B51] Jakobek L. Blesso C. (2024). Beneficial effects of phenolic compounds: native phenolic compounds *vs* metabolites and catabolites. Crit. Rev. Food Sci. Nutr. 64 (25), 9113–9131. 10.1080/10408398.2023.2208218 37140183

[B52] Jean W. H. Lin Y. C. Ang P. Y. Goto K. Lin C. A. Dewi L. (2024). Senolytic effects of exercise in human muscles require acute inflammation. Aging-Us 16 (10), 8599–8610. 10.18632/aging.205827 38752873 PMC11164480

[B53] Jia D. Tian Z. Wang R. (2023). Exercise mitigates age-related metabolic diseases by improving mitochondrial dysfunction. Ageing Res. Rev. 91, 102087. 10.1016/j.arr.2023.102087 37832607

[B54] Kankaanpaeae A. Tolvanen A. Joensuu L. Waller K. Heikkinen A. Kaprio J. (2025). The associations of long-term physical activity in adulthood with later biological ageing and all-cause mortality - a prospective twin study. Eur. J. Epidemiol. 40 (1), 107–122. 10.1007/s10654-024-01200-x 39821867 PMC11799114

[B55] Kessas K. Lounis W. Chouari Z. Vejux A. Lizard G. Kharoubi O. (2024). Benefits of rutin on mitochondrial function and inflammation in an aluminum-induced neurotoxicity rat model: potential interest for the prevention of neurodegeneration. Biochimie 222, 1–8. 10.1016/j.biochi.2024.02.010 38408719

[B56] Kong F. Xie C. Zhao X. Zong X. Bu L. Zhang B. (2022). Resveratrol regulates PINK1/Parkin-mediated mitophagy *via* the lncRNA ZFAS1-miR-150-5p-PINK1 axis, and enhances the antitumor activity of paclitaxel against non-small cell lung cancer. Toxicol. Res. 11 (6), 962–974. 10.1093/toxres/tfac072 36569479 PMC9773061

[B57] Konig T. McBride H. M. (2024). Mitochondrial-derived vesicles in metabolism, disease, and aging. Cell Metab. 36 (1), 21–35. 10.1016/j.cmet.2023.11.014 38171335

[B58] Kopp W. (2024). Aging and “Age-Related” diseases - what is the relation? Aging Dis. 16, 1316–1346. 10.14336/ad.2024.0570 39012663 PMC12096902

[B59] Kramer D. J. Johnson A. A. (2024). Apigenin: a natural molecule at the intersection of sleep and aging. Front. Nutr. 11, 1359176. 10.3389/fnut.2024.1359176 38476603 PMC10929570

[B60] Kroemer G. Maier A. B. Cuervo A. M. Gladyshev V. N. Ferrucci L. Gorbunova V. (2025). From geroscience to precision geromedicine: understanding and managing aging. Cell 188 (8), 2043–2062. 10.1016/j.cell.2025.03.011 40250404 PMC12037106

[B61] Kulkarni H. Gaikwad A. B. (2025). The mitochondria-gut microbiota crosstalk - a novel frontier in cardiovascular diseases. Eur. J. Pharmacol. 998, 177562. 10.1016/j.ejphar.2025.177562 40157703

[B62] Li Z. Zhu J. Wan Z. Li G. Chen L. Guo Y. (2021). Theaflavin ameliorates renal ischemia/reperfusion injury by activating the Nrf2 signalling pathway *in vivo* and *in vitro* . Biomed. Pharmacother. 134, 111097. 10.1016/j.biopha.2020.111097 33341051

[B63] Li L. Lei X. Chen L. Ma Y. Luo J. Liu X. (2024a). Protective mechanism of quercetin compounds against acrylamide-induced hepatotoxicity. Food Sci. Hum. Wellness 13 (1), 225–240. 10.26599/fshw.2022.9250019

[B64] Li Z. Chen L. Qu L. Yu W. Liu T. Ning F. (2024b). Potential implications of natural compounds on aging and metabolic regulation. Ageing Res. Rev. 101, 102475. 10.1016/j.arr.2024.102475 39222665

[B65] Li Z. Kanwal R. Yue X. Li M. Xie A. (2024c). Polyphenols and intestinal microorganisms: a review of their interactions and effects on human health. Food Biosci. 62, 105220. 10.1016/j.fbio.2024.105220

[B66] Lin Q. R. Jia L. Q. Lei M. Gao D. Zhang N. Sha L. (2024). Natural products as pharmacological modulators of mitochondrial dysfunctions for the treatment of diabetes and its complications: an update since 2010. Pharmacol. Res. 200, 107054. 10.1016/j.phrs.2023.107054 38181858

[B67] Lin B. Wu T. Nasb M. Li Z. Chen N. (2025). Regular exercise alleviates metabolic dysfunction-associated steatohepatitis through rescuing mitochondrial oxidative stress and dysfunction in liver. Free Radic. Biol. Med. 230, 163–176. 10.1016/j.freeradbiomed.2025.02.017 39954868

[B68] Liu S. Sui Q. Zou J. Zhao Y. Chang X. (2019). Protective effects of hawthorn (*Crataegus pinnatifida*) polyphenol extract against UVB-induced skin damage by modulating the p53 mitochondrial pathway *in vitro* and *in vivo* . J. Food Biochem. 43 (2), e12708. 10.1111/jfbc.12708 31353662

[B69] Liu C. Hua L. Xin Z. (2024). Synergistic impact of 25-hydroxyvitamin D concentrations and physical activity on delaying aging. Redox Biol. 73, 103188. 10.1016/j.redox.2024.103188 38740004 PMC11103937

[B70] Liu Y. Zhao D. Yang F. Ye C. Chen Z. Chen Y. (2024). *In situ* self-assembled phytopolyphenol-coordinated intelligent nanotherapeutics for multipronged management of ferroptosis-driven Alzheimer’s disease. Acs Nano 18 (11), 7890–7906. 10.1021/acsnano.3c09286 38445977

[B71] Liu H. Song Y. Wang H. Zhou Y. Xu M. Xian J. (2025). Deciphering the power of resveratrol in mitophagy: from molecular mechanisms to therapeutic applications. Phytotherapy Res. 39 (3), 1319–1343. 10.1002/ptr.8433 39754508

[B72] Liu K. Xing S. Abd El-Aty A. M. Tan M. (2025). Precision nutrition based on food bioactive components assisted by delivery nanocarriers for ocular health. Trends Food Sci. Technol. 157, 104923. 10.1016/j.tifs.2025.104923

[B73] Liu Y. Zhou R. Guo Y. Hu B. Xie L. An Y. (2025). Muscle-derived small extracellular vesicles induce liver fibrosis during overtraining. Cell Metab. 37 (4), 824–841.e8. 10.1016/j.cmet.2024.12.005 39879982

[B74] Lopez-Otin C. Blasco M. A. Partridge L. Serrano M. Kroemer G. (2013). The hallmarks of aging. Cell 153 (6), 1194–1217. 10.1016/j.cell.2013.05.039 23746838 PMC3836174

[B75] Lu X. Chen Y. Shi Y. Shi Y. Su X. Chen P. (2025). Exercise and exerkines: mechanisms and roles in anti-aging and disease prevention. Exp. Gerontol. 200, 112685. 10.1016/j.exger.2025.112685 39818278

[B76] Lu Y. Wang K. Hu L. (2025). Advancements in delivery systems for dietary polyphenols in enhancing radioprotection effects: challenges and opportunities. npj Sci. Food 9 (1), 51. 10.1038/s41538-025-00419-6 40229284 PMC11997175

[B77] Ma Z. Cen Y. Xun W. Mou C. Yu J. Hu Y. (2025). Exercise enhances cardiomyocyte mitochondrial homeostasis to alleviate left ventricular dysfunction in pressure overload induced remodelling. Sci. Rep. 15 (1), 11698. 10.1038/s41598-025-95637-z 40188200 PMC11972341

[B78] Manford A. G. Rodriguez-Perez F. Shih K. Y. Shi Z. Berdan C. A. Choe M. (2020). A cellular mechanism to detect and alleviate reductive stress. Cell 183 (1), 46–61.e21. 10.1016/j.cell.2020.08.034 32941802

[B79] Mao Y. Huang J. Li S. Chen G. Du Y. Kang M. (2025). Brussels chicory enhances exhaustive aerobic exercise performance and post-exercise recovery, possibly through promotion of lactate oxidation: a pilot randomized, single-blind, placebo-controlled, two-way crossover study. Nutrients 17 (2), 365. 10.3390/nu17020365 39861495 PMC11769108

[B80] Marchi S. Guilbaud E. Tait S. W. G. Yamazaki T. Galluzzi L. (2023). Mitochondrial control of inflammation. Nat. Rev. Immunol. 23 (3), 159–173. 10.1038/s41577-022-00760-x 35879417 PMC9310369

[B81] Martini H. Passos J. F. (2023). Cellular senescence: all roads lead to mitochondria. Febs J. 290 (5), 1186–1202. 10.1111/febs.16361 35048548 PMC9296701

[B82] Matsumura Y. Kitabatake M. Kayano S. I. Ito T. (2023). Dietary phenolic compounds: their health benefits and association with the gut microbiota. Antioxidants 12 (4), 880. 10.3390/antiox12040880 37107256 PMC10135282

[B83] Mehrabi A. Nuori R. Gaeini A. Amirazodi M. Mehrtash M. Esfahlani M. A. (2025). The antiaging and antioxidative effects of a combination of resveratrol and high-intensity interval training on the frontal lobe in aged rats: the role of SIRTS 4, SIRTS 5, SOD1, and SOD2. Oxidative Med. Cell. Longev. 2025, 8251896. 10.1155/omcl/8251896 39959582 PMC11824298

[B84] Michaudel C. Sokol H. (2020). The gut microbiota at the service of immunometabolism. Cell Metab. 32 (4), 514–523. 10.1016/j.cmet.2020.09.004 32946809

[B85] Millar C. L. Iloputaife I. Baldyga K. Norling A. M. Boulougoura A. Vichos T. (2025). A pilot study of senolytics to improve cognition and mobility in older adults at risk for Alzheimer’s disease. EBioMedicine 113, 105612. 10.1016/j.ebiom.2025.105612 40010154 PMC11907475

[B86] Min L. Ablitip A. Wang R. Luciana T. Wei M. Ma X. (2024). Effects of exercise on gut microbiota of adults: a systematic review and meta-analysis. Nutrients 16 (7), 1070. 10.3390/nu16071070 38613103 PMC11013040

[B87] Mollazadeh H. Tavana E. Fanni G. Bo S. Banach M. Pirro M. (2021). Effects of statins on mitochondrial pathways. J. Cachexia Sarcopenia Muscle 12 (2), 237–251. 10.1002/jcsm.12654 33511728 PMC8061391

[B88] Moradi N. Sanfrancesco V. C. Champsi S. Hood D. A. (2024). Regulation of lysosomes in skeletal muscle during exercise, disuse and aging. Free Radic. Biol. Med. 225, 323–332. 10.1016/j.freeradbiomed.2024.09.028 39332541

[B89] Murley A. Popovici A. C. Hu X. S. Lund A. Wickham K. Durieux J. (2025). Quiescent cell re-entry is limited by macroautophagy-induced lysosomal damage. Cell 188, 2670–2686.e14. 10.1016/j.cell.2025.03.009 40203825

[B90] Nakai R. Varnum S. Field R. L. Shi H. Giwa R. Jia W. (2024). Mitochondria transfer-based therapies reduce the morbidity and mortality of Leigh syndrome. Nat. Metab. 6 (10), 1886–1896. 10.1038/s42255-024-01125-5 39223312 PMC12188917

[B91] Nature (2010). Npre.2010.4421.1. Available online at: https://www.nature.com/articles/npre.2010.4421.1 (Accessed August 14, 2025).

[B92] Perez-Jimenez J. Agnant K. Lamuela-Raventos R. M. St-Onge M. P. (2023). Dietary polyphenols and sleep modulation: current evidence and perspectives. Sleep. Med. Rev. 72, 101844. 10.1016/j.smrv.2023.101844 37659249 PMC10872761

[B93] Pluta R. Furmaga-Jablonska W. Januszewski S. Czuczwar S. J. (2022). Post-ischemic brain neurodegeneration in the form of Alzheimer’s disease proteinopathy: possible therapeutic role of curcumin. Nutrients 14 (2), 248. 10.3390/nu14020248 35057429 PMC8779038

[B94] Qin S. Chi X. Zhu Z. Chen C. Zhang T. He M. (2025). Oocytes maintain low ROS levels to support the dormancy of primordial follicles. Aging Cell 24 (1), e14338. 10.1111/acel.14338 39297300 PMC11709087

[B95] Qu Q. Chen Y. Wang Y. Long S. Wang W. Yang H. Y. (2024). Lithocholic acid phenocopies anti-ageing effects of calorie restriction. Nature 643, 192–200. 10.1038/s41586-024-08329-5 39695227 PMC12222012

[B96] Ravindran R. Gustafsson a. B. (2025). Mitochondrial quality control in cardiomyocytes: safeguarding the heart against disease and ageing. Nat. Rev. Cardiol. 22, 798–813. 10.1038/s41569-025-01142-1 40113864

[B97] Redza-Dutordoir M. Averill-Bates D. A. (2021). Interactions between reactive oxygen species and autophagy special issue: death mechanisms in cellular homeostasis. Biochimica Biophysica Acta-Molecular Cell Res. 1868 (8), 119041. 10.1016/j.bbamcr.2021.119041 33872672

[B98] Ren L. Hu F. Walsh S. Jin X. Hu Y. Li S. (2025). Healthy lifestyle factors outweigh influence of APOE genetic risk on extending cognitively healthy life expectancy among Chinese older adults: evidence from a nationwide cohort study. Alzheimer’s Dementia 21 (4), e70090. 10.1002/alz.70090 40226865 PMC11995295

[B99] Rocchetti G. Gregorio R. P. Lorenzo J. M. Barba F. J. Garcia Oliveira P. Prieto M. A. (2022). Functional implications of bound phenolic compounds and phenolics-food interaction: a review. Compr. Rev. Food Sci. Food Saf. 21 (2), 811–842. 10.1111/1541-4337.12921 35150191

[B100] Rubilar J. C. Outeiro T. F. Klein A. D. (2024). The lysosomal β-glucocerebrosidase strikes mitochondria: implications for Parkinson’s therapeutics. Brain 147 (8), 2610–2620. 10.1093/brain/awae070 38437875

[B101] Russo L. Babboni S. Andreassi M. G. Daher J. Canale P. Del Turco S. (2025). Treating metabolic dysregulation and senescence by caloric restriction: killing two birds with one stone? Antioxidants 14 (1), 99. 10.3390/antiox14010099 39857433 PMC11763027

[B102] Ryu K. W. Fung T. S. Baker D. C. Saoi M. Park J. Febres-Aldana C. A. (2024). Cellular ATP demand creates metabolically distinct subpopulations of mitochondria. Nature 635 (8039), 746–754. 10.1038/s41586-024-08146-w 39506109 PMC11869630

[B103] Safdar A. Saleem A. Tarnopolsky M. A. (2016). The potential of endurance exercise-derived exosomes to treat metabolic diseases. Nat. Rev. Endocrinol. 12 (9), 504–517. 10.1038/nrendo.2016.76 27230949

[B104] Sarkar J. Das M. Howlader M. S. I. Prateeksha P. Barthels D. Das H. (2022). Epigallocatechin-3-gallate inhibits osteoclastic differentiation by modulating mitophagy and mitochondrial functions. Cell Death Dis. 13 (10), 908. 10.1038/s41419-022-05343-1 36307395 PMC9616829

[B105] Schuster C. Wolpert N. Moustaid-Moussa N. Gollahon L. S. (2022). Combinatorial effects of the natural products arctigenin, chlorogenic acid, and cinnamaldehyde commit oxidation assassination on breast cancer cells. Antioxidants 11 (3), 591. 10.3390/antiox11030591 35326241 PMC8945099

[B106] Sen P. Shah P. P. Nativio R. Berger S. L. (2016). Epigenetic mechanisms of longevity and aging. Cell 166 (4), 822–839. 10.1016/j.cell.2016.07.050 27518561 PMC5821249

[B107] ShaoM. QiuY. ShenM. LiuW. FengD. LuoZ. (2024). Procyanidin C1 inhibits bleomycin-induced pulmonary fibrosis in mice by selective clearance of senescent myofibroblasts. Faseb J. 38 (13), e23749. 10.1096/fj.202302547RR 38953707

[B108] Sharma V. Mehdi M. M. (2023). Oxidative stress, inflammation and hormesis: the role of dietary and lifestyle modifications on aging. Neurochem. Int. 164, 105490. 10.1016/j.neuint.2023.105490 36702401

[B109] Sharma V. K. Singh S. P. Singh B. N. Rao C. V. Barik S. K. (2022). Nanocurcumin potently inhibits SARS-CoV-2 spike protein-induced cytokine storm by deactivation of MAPK/NF-κB signaling in epithelial cells. ACS Appl. Bio Mater. 5 (2), 483–491. 10.1021/acsabm.1c00874 35112841

[B110] Singh R. Akhtar N. Haqqi T. M. (2010). Green tea polyphenol epigallocatechin-3-gallate: inflammation and arthritis. [corrected]. Life Sci. 86 (25-26), 907–918. 10.1016/j.lfs.2010.04.013 20462508 PMC3146294

[B111] Son J. M. Lee C. (2021). Aging: all roads lead to mitochondria. Seminars Cell Dev. Biol. 116, 160–168. 10.1016/j.semcdb.2021.02.006 33741252 PMC9774040

[B112] Song Z. Y. Ghochani M. McCaffery J. M. Frey T. G. Chan D. C. (2009). Mitofusins and OPA1 mediate sequential steps in mitochondrial membrane fusion. Mol. Biol. Cell 20 (15), 3525–3532. 10.1091/mbc.E09-03-0252 19477917 PMC2719570

[B113] Stamatakis E. Koemel N. A. Biswas R. K. Ahmadi M. N. Allman-Farinelli M. Trost S. G. (2025). Minimum and optimal combined variations in sleep, physical activity, and nutrition in relation to all-cause mortality risk. Bmc Med. 23 (1), 111. 10.1186/s12916-024-03833-x 40001093 PMC11863424

[B114] Tang C. H. (2021). Strategies to utilize naturally occurring protein architectures as nanovehicles for hydrophobic nutraceuticals. Food Hydrocoll. 112, 106344. 10.1016/j.foodhyd.2020.106344

[B115] Tang Q. Zheng G. Feng Z. Chen Y. Lou Y. Wang C. (2017). Trehalose ameliorates oxidative stress-mediated mitochondrial dysfunction and ER stress *via* selective autophagy stimulation and autophagic flux restoration in osteoarthritis development. Cell Death Dis. 8 (10), e3081. 10.1038/cddis.2017.453 28981117 PMC5680575

[B116] Tang G. Y. Meng X. Gan R. Y. Zhao C. N. Liu Q. Feng Y. B. (2019). Health functions and related molecular mechanisms of tea components: an update review. Int. J. Mol. Sci. 20 (24), 6196. 10.3390/ijms20246196 31817990 PMC6941079

[B117] Tang C. H. Chen H. L. Dong J. R. (2023). Solid lipid nanoparticles (SLNs) and nanostructured lipid carriers (NLCs) as food-grade nanovehicles for hydrophobic nutraceuticals or bioactives. Appl. Sciences-Basel 13 (3), 1726. 10.3390/app13031726

[B118] Tessier A. J. Wang F. Korat A. A. Eliassen A. H. Chavarro J. Grodstein F. (2025). Optimal dietary patterns for healthy aging. Nat. Med. 31, 1644–1652. 10.1038/s41591-025-03570-5 40128348 PMC12092270

[B119] Tribble J. R. Joee M. Varricchio C. Otmani A. Canovai A. Habchi B. (2024). NMNAT2 is a druggable target to drive neuronal NAD production. Nat. Commun. 15 (1), 6256. 10.1038/s41467-024-50354-5 39048544 PMC11269627

[B120] Vaccaro A. Dor Y. K. Nambara K. Pollina E. A. Lin C. Greenberg M. E. (2020). Sleep loss can cause death through accumulation of reactive oxygen species in the gut. Cell, 181(6), 1307–1328.e15. 10.1016/j.cell.2020.04.049 32502393

[B121] Walker E. M. Pearson G. L. Lawlor N. Stendahl A. M. Lietzke A. Sidarala V. (2025). Retrograde mitochondrial signaling governs the identity and maturity of metabolic tissues. Sci. (New York, N.Y.) 388 (6743), eadf2034. 10.1126/science.adf2034 39913641 PMC11985298

[B122] Wan W. Zhu W. Wu Y. Long Y. Liu H. Wan W. (2021). Grape seed proanthocyanidin extract moderated retinal pigment epithelium cellular senescence through NAMPT/SIRT1/NLRP3 pathway. J. Inflamm. Res. 14, 3129–3143. 10.2147/jir.S306456 34285539 PMC8286255

[B123] Wang X. He Y. Tian J. Muhammad I. Liu M. Wu C. (2021). Ferulic acid prevents aflatoxin B1-induced liver injury in rats *via* inhibiting cytochrome P450 enzyme, activating Nrf2/GST pathway and regulating mitochondrial pathway. Ecotoxicol. Environ. Saf. 224, 112624. 10.1016/j.ecoenv.2021.112624 34416636

[B124] Wang R. Lee Y. G. Dhandapani S. Baek N. I. Kim K. P. Cho Y. E. (2023). 8-paradol from ginger exacerbates PINK1/Parkin mediated mitophagy to induce apoptosis in human gastric adenocarcinoma. Pharmacol. Res. 187, 106610. 10.1016/j.phrs.2022.106610 36521573

[B125] Wang T. Sun X. Zhang Y. Wang Q. Cheng W. Gao Y. (2025). Baicalin promotes skeletal muscle fiber remodeling by activating the p38MAPK/PGC-1α signaling pathway. J. Agric. Food Chem. 73 (11), 6878–6889. 10.1021/acs.jafc.5c00300 40103396

[B126] Wang W. Cui Y. Liu H. Wang Y. Nan B. Li X. (2025). Progress in the bioavailability of natural astaxanthin: influencing factors, enhancement strategies, evaluation methods, and limitations of current research. Trends Food Sci. Technol. 160, 104998. 10.1016/j.tifs.2025.104998

[B127] WHO. INT. (2025). Ageing-and-health. Available online at: https://www.who.int/zh/news-room/fact-sheets/detail/ageing-and-health (Accessed August 5, 2025).

[B128] Wu M. Luo Q. Nie R. Yang X. Tang Z. Chen H. (2021). Potential implications of polyphenols on aging considering oxidative stress, inflammation, autophagy, and gut microbiota. Crit. Rev. Food Sci. Nutr. 61 (13), 2175–2193. 10.1080/10408398.2020.1773390 32496818

[B129] Wu Z. Qu J. Zhang W. Liu G.-H. (2024). Stress, epigenetics, and aging unraveling the intricate crosstalk. Mol. Cell 84 (1), 34–54. 10.1016/j.molcel.2023.10.006 37963471

[B130] Xiang Z. Guan H. Zhao X. Xie Q. Xie Z. Cai F. (2024). Dietary gallic acid as an antioxidant: a review of its food industry applications, health benefits, bioavailability, nano-delivery systems, and drug interactions. Food Res. Int. 180, 114068. 10.1016/j.foodres.2024.114068 38395544

[B131] Yoon M. S. (2017). mTOR as a key regulator in maintaining skeletal muscle mass. Front. Physiol. 8, 788. 10.3389/fphys.2017.00788 29089899 PMC5650960

[B132] Yu T. Wang L. Zhang L. Deuster P. A. (2023). Mitochondrial fission as a therapeutic target for metabolic diseases: insights into antioxidant strategies. Antioxidants 12 (6), 1163. 10.3390/antiox12061163 37371893 PMC10295595

[B133] Yuan S. Kuai Z. Zhao F. Xu D. Wu W. (2025). Improving effect of physical exercise on heart failure: reducing oxidative stress-induced inflammation by restoring Ca^2+^ homeostasis. Mol. Cell. Biochem. 480 (4), 2471–2486. 10.1007/s11010-024-05124-8 39365389

[B134] Zhan J. Yan Z. Zhao M. Qi W. Lin J. Lin Z. (2020). Allicin inhibits osteoblast apoptosis and steroid-induced necrosis of femoral head progression by activating the PI3K/AKT pathway. Food Funct. 11 (9), 7830–7841. 10.1039/d0fo00837k 32808945

[B135] Zhang T. G. Miao C. Y. (2023). Mitochondrial transplantation as a promising therapy for mitochondrial diseases. Acta Pharm. Sin. B 13 (3), 1028–1035. 10.1016/j.apsb.2022.10.008 36970208 PMC10031255

[B136] Zhang Z. D. Milman S. Lin J. R. Wierbowski S. Yu H. Barzilai N. (2020). Genetics of extreme human longevity to guide drug discovery for healthy ageing. Nat. Metab. 2 (8), 663–672. 10.1038/s42255-020-0247-0 32719537 PMC7912776

[B137] Zhang X. Jiang Y. Mao J. Ren X. Ji Y. Mao Y. (2021). Hydroxytyrosol prevents periodontitis-induced bone loss by regulating mitochondrial function and mitogen-activated protein kinase signaling of bone cells. Free Radic. Biol. Med. 176, 298–311. 10.1016/j.freeradbiomed.2021.09.027 34610362

[B138] Zhang L. Liu Y. Sun Y. Zhang X. (2022). Combined physical exercise and diet: regulation of gut microbiota to prevent and treat of metabolic disease: a review. Nutrients 14 (22), 4774. 10.3390/nu14224774 36432462 PMC9699229

[B139] Zhao X. Wang C. Dai S. Liu Y. Zhang F. Peng C. (2022). Quercetin protects ethanol-induced hepatocyte pyroptosis *via* scavenging mitochondrial ROS and promoting PGC-1α-Regulated mitochondrial homeostasis in L02 cells. Oxidative Med. Cell. Longev. 2022, 4591134. 10.1155/2022/4591134 35879991 PMC9308520

